# Dual functional highly luminescence B, N Co-doped carbon nanodots as nanothermometer and Fe^3+^/Fe^2+^ sensor

**DOI:** 10.1038/s41598-020-59958-5

**Published:** 2020-02-20

**Authors:** Lazo Jazaa Mohammed, Khalid M. Omer

**Affiliations:** Department of Chemistry, College of Science, University of Sulaimani, Qliasan St, Sulaimani City, Kurdistan Region Iraq

**Keywords:** Analytical chemistry, Materials chemistry

## Abstract

Dual functional fluorescence nanosensors have many potential applications in biology and medicine. Monitoring temperature with higher precision at localized small length scales or in a nanocavity is a necessity in various applications. As well as the detection of biologically interesting metal ions using low-cost and sensitive approach is of great importance in bioanalysis. In this paper, we describe the preparation of dual-function highly fluorescent B, N-co-doped carbon nanodots (CDs) that work as chemical and thermal sensors. The CDs emit blue fluorescence peaked at 450 nm and exhibit up to 70% photoluminescence quantum yield with showing excitation-independent fluorescence. We also show that water-soluble CDs display temperature-dependent fluorescence and can serve as highly sensitive and reliable nanothermometers with a thermo-sensitivity 1.8% °C^−1^, and wide range thermo-sensing between 0–90 °C with excellent recovery. Moreover, the fluorescence emission of CDs are selectively quenched after the addition of Fe^2+^ and Fe^3+^ ions while show no quenching with adding other common metal cations and anions. The fluorescence emission shows a good linear correlation with concentration of Fe^2+^ and Fe^3+^ (R^2^ = 0.9908 for Fe^2+^ and R^2^ = 0.9892 for Fe^3+^) with a detection limit of of 80.0 ± 0.5 nM for Fe^2+^ and 110.0 ± 0.5 nM for Fe^3+^. Considering the high quantum yield and selectivity, CDs are exploited to design a nanoprobe towards iron detection in a biological sample. The fluorimetric assay is used to detect Fe^2+^ in iron capsules and total iron in serum samples successfully.

## Introduction

Improvements in creating innovative sensors for detecting multiple parameters have grown much attention because they are more proficient than the sensors for a single objective^1^. Currently, various fluorescent sensors for the simultaneous detection of two or more analytes or other parameters such as pH, temperature, and UV-light have been reported^2–6^. Temperature is an essential thermodynamic variable that affects biochemical and physiological processes intensely^7^. High-precision determination of temperature is of infinite importance owing to the widespread applications in human life, research studies, and industrial fields. An optical temperature sensor has the benefits of contactless detection, a high-temperature resistance, a diverse temperature range, not interfering with the original temperature, and prompt response^8^. Some classes of luminescent nanomaterials such as, quantum dots, carbon dots, polymer dots, and nanodiamonds displayed temperature-dependent luminescence^9–11^. Carbon dots take the lead as temperature detectors establishing great potential as a probe for the detection of temperature in complicated environments such as biological media due to their unique properties and small size comparing to the other nanomaterials^6,12–14^.

Iron homeostasis disorders are one of the utmost regular diseases of humans and cover an expansive range of diseases with various signs and symptoms, starting from anemia to excesses of iron, liver and kidney diseases, diabetes Mellitus, cardiovascular disease and very likely to neurodegenerative disorders^15,16^. The most common oxidation states of iron are Fe^3+^ and Fe^2+^ vigorously altering to one another, which makes it challenging to detect Fe^3+^ or Fe^2+^ ions only^17^. However, considering an easy, precise, and field-appropriate detection method for total iron (Fe^3+^ and/or Fe^2+^ ions) probing is by far of great importance for the analysis of iron elaborating in medical diagnosis, environmental monitoring of water quality and other quality control examinations^17–19^. The most common method for the detection of iron ions includes voltammetry, electron paramagnetic resonance, spectrophotometry, atomic absorption spectrometry, inductively coupled plasma-atomic emission spectroscopy and inductively coupled plasma mass spectrometry. The mentioned methods need complex instrumentation, tedious sample preparation, lengthy procedures, and experienced personnel that restricts their routine analysis and are inapplicable for most laboratories^17,19–21^. Among all detection methods, fluorescence spectrometry has gained much attention and is a dominant optical method for trace analysis of significant biological samples because of its high sensitivity, simple operation, being reproducible and rapid implementation^22^.

Carbon dots are a class of advanced fluorescent nanomaterials, that are becoming ever more favored lately for their facile fabrication, large-scale production, good biocompatibility, low toxicity, and stability^23,24^. Additionally, owing to their capability of up-conversion and down-conversion, superiority in photostability, fluorescence non-blinking emission and environment friendliness in comparison to organic dyes or semiconductor quantum dots (QDs) or metal nanoparticles, CDs are more appropriate to be used as fluorescent nanosensors by an increase in fluorescence emissions or quenching^25,26^. Thus, great attention has been paid to investigate various methods for the synthesis of carbon dots, such as chemical oxidation of carbon resources, laser ablation, microwave-assisted method, ultrasonic production, pyrolysis, electrochemical etching, and hydrothermal method^27^. Among these preparation methods, the hydrothermal carbonization of small molecules is an outstanding method to produce CDs, because of mild conditions and deprived of concentrated acids.

The size and functional groups on the surface of carbon dots can be wisely controlled to accomplish improved performance as intended in any specific application. Particularly, the CDs emissions could be modified by adjusting the condensation reaction, chemical manipulations, or by doping with other elements. Changes in the composition, type, and a number of the surface functional groups can influence the fluorescence quantum yields and chemical reactivity of the CDs. Hence by choosing suitable precursors, solvents, heating temperature, and heating time, the surface functional groups and the photoluminescence properties could be modified^28–32^. Lately, heteroatom-doped CDs have been scrutinized, and it is reported that heteroatom doping significantly affects the properties of the resultant CD materials^33,34^. Nitrogen is utterly the most noticeable dopant, whereas other dopants like boron (B), sulfur (S) and phosphorus (P) were co-doped with nitrogen^35^. Thus, engineering of the surface state of carbon dots is the key issue to obtain dual functional mode properties.

In literature, reports on thermo-sensing with detection of iron ions are scarce. Cui *et al*. reported dual functional carbon dots for the detection of only one oxidation state of iron (Fe^3+^) with thermal-sensing^36^. To the best of our knowledge, no comprehensive work was dedicated to engineer CDs with dual functionality towards both of the two oxidation states of iron and temperature. Our work aims at filling this gap and engineer a co-doped carbon nanodots to function dually towards iron ions and temperature.

**Figure 1:**
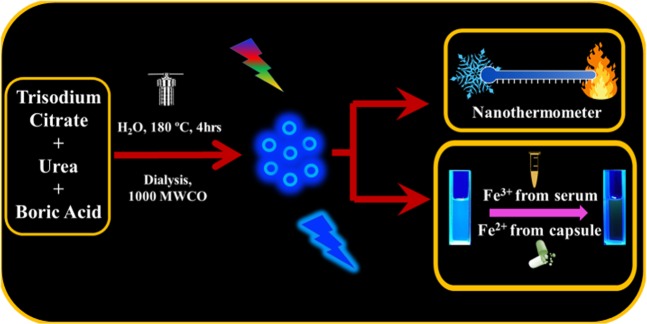
Schematic illustration showing the preparation and dual function routes of the CDs’ emission by iron ions and temperature.

Here, we introduce a simple, low-cost and green synthetic strategy to prepare the water-soluble, highly fluorescent nitrogen and boron co-doped carbon dots. The preparation strategy is the single-step hydrothermal treatment of tri-precursor of trisodium citrate, urea, and boric acid. The prepared CDs show great sensitivity and reversibility towards temperature changes in the range of 20 °C to 90 °C. Furthermore, the blue fluorescence could be specifically quenched by each Fe^3+^ and Fe^2+^ ions individually. The fluorimetric assay was developed for the analysis of Fe^3+^ and/or Fe^2+^ ions. Successively, the practical applications of the established assays for real samples like human serum and pharmaceutical dosage form were validated. Figure 1 shows a diagram of the preparation and detection routes of CDs.

## Experimental Section

### Chemicals and materials

For all experiments, double deionized water was used. All chemicals used were of analytical reagent grade and used as received with no further purification, obtained from commercial sources. Urea, trisodium citrate, and boric acid were purchased from Sigma-Aldrich. Fluorescein was purchased from Sigma-Aldrich, Ethanol (99.8%), hydrochloric acid (36–38%), Nitric acid (68–70%), NaOH, (NH_4_)_2_Fe(SO_4_), Fe(NO_3_)_3_, Mg(NO_3_)_2_, Al(NO_3_)_3_, Cu(NO_3_)_2_, Zn(NO_3_)_2_, Pb(NO_3_)_2_, NiSO_4_, AgNO_3_, CrCl_3_, MnCl_2_, Cd(NO_3_)_2_, HgCl_2_, Co(NO_3_)_2_, NaCl, Na_2_SO_4_, Na_2_CO_3_, KI, KBr, Na_2_S, CH_3_COONa, D-glucose, Glycine, and Creatinine were purchased from Sigma-Aldrich. The sera were from regular patients collected from Pediatric Teaching Hospital in the Sulaimani City, Kurdistan Region, Iraq.

### Preparation of carbon dots

The co-doped CDs were synthesized by conjoining both reported methods with minor modifications^24,37^ Briefly, 0.2 g trisodium citrate, 0.2 g urea, and 0.2 g boric acid were added to 10 mL water in a Teflon equipped stainless steel autoclave, followed by hydrothermal treatment at 180 °C for 4 h. Then the autoclave was cooled down to room temperature under ambient condition and the CDs were further purified through a dialysis tube (1000 Da, molecular weight cutoff) for about 1 h. The final solution needed extra dilution because of its high intensity and high quantum yield, 1 mL (13 mg.mL^−1^) of CD stock solution was diluted to 100 mL for further characterization and application.

### Stability of CDs

HCl or NaOH was used to adjust the pH of the resultant CD solution. Since CD solutions with pH lower than 3 show very low or no fluorescence intensity, a series of CD solutions with pH from 3 to 10 were prepared keeping the final concentration the same for all. Then, the fluorescence spectra of the solutions were carried out.

The fluorescence spectra of CDs were recorded in different concentrations of NaCl (0.1. 0.2, 0.3, 0.4, 0.5, 0.6, 0.7, 0.8, 0.9 and 1 M). For the photostability of CDs, a sample of CD solution was kept under UV-light for 4 hours and fluorescence spectra were recorded every 15 minutes.

### Characterization

UV-Vis absorption spectra were recorded on a Cary 60 Spectrophotometer (Agilent Technologies, USA). FTIR spectra were obtained on Cary 660 ATR-FTIR connected to ATR (Agilent technologies, USA). Photoluminescence spectra were recorded on Cary Eclipsed Fluorescence Spectrophotometer (Agilent Technologies, USA). High-resolution transmission electron microscopy (HR-TEM) was applied to observe the morphology and size of CDs, which was carried out using TECNAI G2 F20 microscope (Ames Lab, USA) at 200 kV. The surface and core chemical composition of the CDs were examined by X-ray photoelectron spectroscopy (XPS) using Thermo Escalab 250 XI (Thermo Scientific, USA). The Raman spectra were generated using inVia Reflex (Renishaw, UK) with an Nd:YAG laser source at 785 nm. X-ray diffraction (XRD spectra were obtained on Empyrean X-ray diffractometer, (PANalytical, Netherland). Samples for XRD were prepared by casting few drops of CDs on a glass slide, then drying by a heater with temperature 60 °C.

### Determination of the quantum yields

The fluorescence quantum yield ($\varnothing$) of CDs was calculated from the following equation using fluorescein as the standard, whose quantum yield is about 0.95.

$$\varnothing =\varnothing s\frac{F}{Fs}\frac{As}{A}\frac{{\eta }^{2}}{\eta {s}^{2}}$$

where $\varnothing$ is the quantum yield, F and Fs are the integrated fluorescent intensity, A and As are the absorbance, η and ηs represent the refractive index of the solvent of CDs and fluorescein solution, respectively. The absorbance was measured at 340 nm, and the fluorescence spectrum was obtained at the excitation wavelength of 340 nm.

### Fluorescence assay for iron ions

100 μL of CD solution was mixed with 3 mL of different concentrations of iron solutions. The pH of each solution was adjusted to 5.0 with HCl or NaOH. The fluorescence spectra were recorded in 5 min after mixing.

### Analysis of Fe^2+^ in capsules

10 capsules (purchased from Sulaimani City pharmaceutical stores) were mixed and grounded to make a homogeneous powder mixture. 1.0 g from the powder was dissolved in 15–20 mL of 6.0 M HCl and then heated for 15 min with stirring. The solution was filtered to get rid of non-soluble particles. Finally, the clear solution was completed to 100 mL with DI water for the fluorescence measurement.100 μL of carbon dots solution was mixed with 1 mL of capsule sample and different concentrations of Fe^2+^ standard solutions were added. The content of the added Fe^2+^ in capsule samples was analyzed using the developed sensing technique and the recovery efficiency was calculated.

### Detection of Fe^3+^ in serum

For the analysis of Fe^3+^ in human serum, pretreatment of the sera is required to remove proteins and release Fe^3+^, this step is called deproteinization. After mixing equal volumes of the serum and ethanol, the resulting solution was heated to 95 °C for 15 min, then cooled down and centrifuged at 10000 rpm for 20 min and the clear supernatant was collected. To ensure the entire Fe element existed as a free ferric ion state, 1 mL concentrated 6 M HNO_3_ was added as an oxidizing agent to the supernatant. In the analytical assay, 100 μL of CD solution was mixed with different volumes of deproteinized human serum and then analyzed with the proposed method. For quantification of the unknown amount of Fe^3+^ in human serum, the standard addition method with Fe(NO_3_)_3_ as the standard was performed. The deproteinized human serum samples were first spiked with Fe^3+^ at different concentrations levels and then measured using the doped carbon dots.

To calculate the recovery percentage of our method, we used the results from a clinical fully automatic biochemical analyzer (Cobas c311) as the standard protocol which uses photometry for the determination of total Iron in serum. Under acidic conditions, iron is liberated from transferrin. Lipemic samples are clarified by the detergent. Ascorbate reduces the released Fe^3+^ ions to Fe^2+^ ions which then react with FerroZine to form a colored complex. The color intensity is directly proportional to the iron concentration and can be measured photometrically^38^.

### Thermosensing experiment

The temperature of the CD solution was adjusted at various temperatures (0, 10, 20, 30, 40, 50, 60, 70, 80 and 90 °C) using a water bath. The duration time of the CD solutions at each temperature was 10 minutes, then the fluorescence spectra were recorded directly. The temperature change before and after the fluorescence measurement was ±1.0.

## Results and Discussion

**Figure 2:**
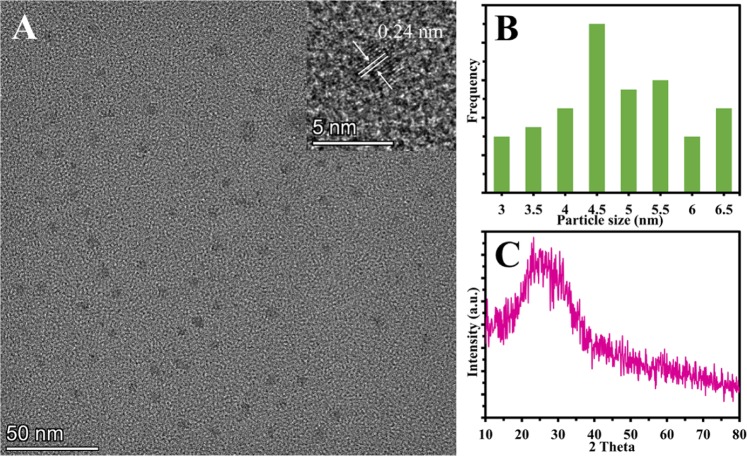
(**A**) TEM images of CDs. (inset is the HR-TEM image of an individual BNCD). (**B**) Size distribution histogram based on counting 70 nanoparticles of CDs. (**C**) XRD spectrum of CDs.

The size and morphology of the CDs were assessed using the transmission electron microscopic (TEM) image. As in Fig. 2A,B, showing that the CDs have a size distribution extending from 3 to 6.5 nm and from the histogram it is proven that the CDs have an average diameter of 4.5 nm. The high-resolution transmission electron microscopy (HRTEM) reveals the existence of a lattice structure in BNCDs (inset of Fig. 2A). The lattice spacing is measured to be 0.24 nm, corresponding to the (020) plane of graphite^39,40^.

Powder X-ray diffraction (XRD) of CDs indicates an amorphous nature because of highly disordered carbon atoms^41^. The XRD configuration belonging to the prepared CDs (Fig. 2C) displays a wide peak positioned at about 2θ = 25° which is in accordance with most of the reported studies clarifying the amorphous carbon phase^30,42^.

**Figure 3:**
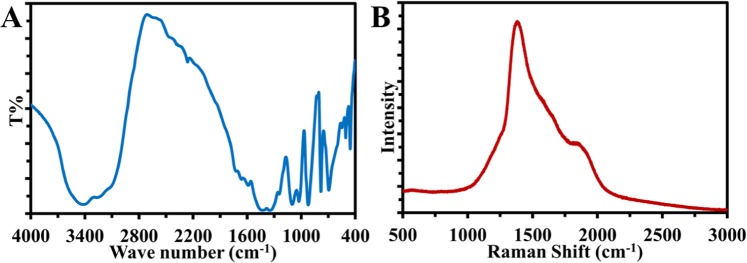
(**A**) FTIR spectrum of CDs. (**B**) Raman spectrum of CDs.

In the FT-IR spectrum in (Fig. 3A), the absorption band between 3100 cm^−1^ and 3600 cm^−1^ is attributed to the O–H and N–H bonds^43,44^. Carboxylic C=O bonds are shown at 1600–1750 cm^−1^ ^43^ which point to that the citrate molecules have been adsorbed on the surface of the particles, these groups are responsible for making the CDs hydrophilic and dissolvable in water and also increase their stability^44^. The peak at 1450 cm^−1^ is assigned for B–O stretching vibration^45,46^ and at 1030 cm^−1^ for B–O–C^45,46^ the two peaks at 1360 cm^−1^ & 790 cm^−1^ contribute to sp^2^-bonded B–N (in-plane B–N stretching vibration, out-plane bending vibration respectively)^37,45^ Whereas the peak at 1100 cm^−1^ to sp^3^-bonding^37,45^.

As shown in Fig. 3B the Raman spectrum of the CDs exhibit a peak with high intensity at around 1380.5 cm^−1^ attributing to the D-band (sp^3^ hybridization)^47–49^ while the shoulder at 1596.6 cm^−1^ represents the G band (sp^2^ hybridized)^50,51^ but the broadening is because of the incorporation of oxygen-containing functional groups, B and N elements in the CD matrix^52^. And there is a shoulder located at 1865.1 cm^−1^, which represents D’ peak that is associated with the presence of N dopants in the lattice^53^. Raman spectrum confirms the doping of nitrogen and boron in the CD entities.

**Figure 4:**
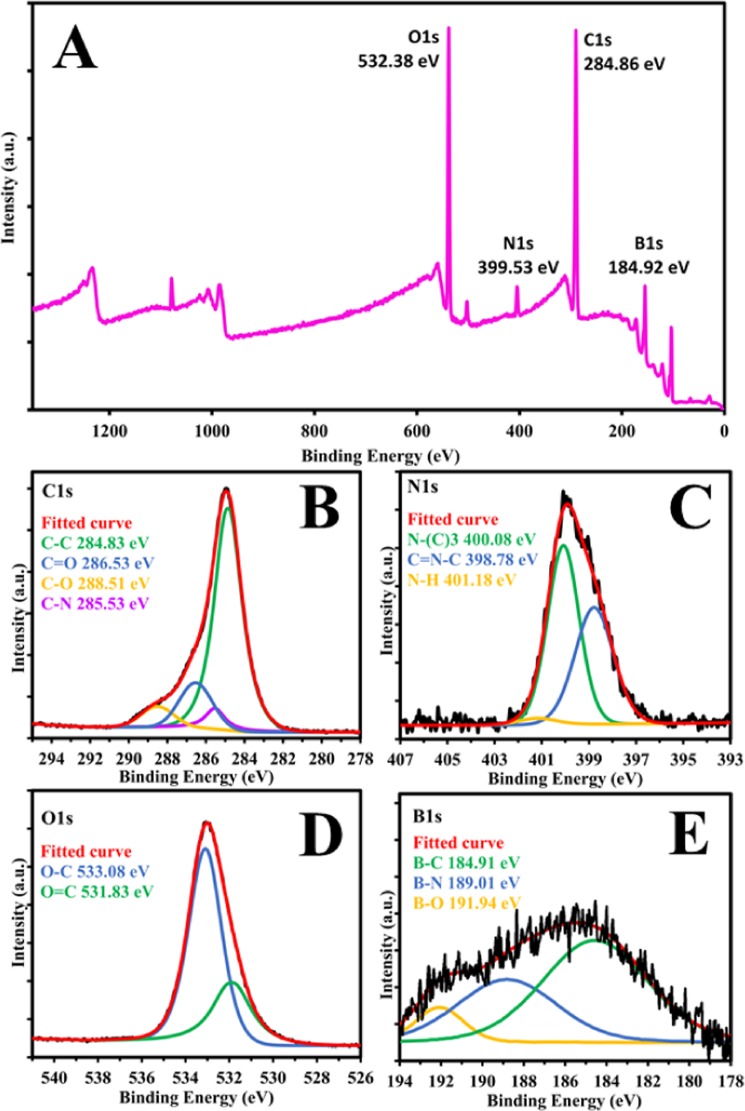
High-resolution XPS spectra of CDs. (**B**) Survey spectra. (**B**) C1s. (**C**) N1s. (**D**) O1s and (**E**) B1s.

X-ray photoelectron spectroscopy (XPS) was carried out to further investigate the surface composition and doping of nitrogen and boron in the CDs (Fig. 4). The XPS survey spectra shown in Fig. 4A displays the characteristic peaks: O1s (532.38 eV), N1s (399.53 eV), C1s (284.86 eV), and B1s (184.92 eV). C 1s Spectrum (Fig. 4B) shows four characteristic peaks at 284.83 eV, 286.53 eV, 288.51 eV, and 285.53 eV corresponding to C-C, C=O, C-O and C-N respectively^19,52^. N 1s spectrum (Fig. 4C) shows three main peaks at 400.08 eV, 398.78 eV and 401.18 eV which correspond to N-(C)3, C=N-C and N-H respectively^54^. O 1s high-resolution spectrum (Fig. 4D) was fitted with two major peaks at 533.08 eV and 531.83 eV which can be assigned to O-C and O=C respectively^55^. The high-resolution B1s spectrum of the CDs (Fig. 4E) reveals three peaks at 184.91 eV, 189.01 eV and 191.94 eV which are attributed to B-C, B-N and B-O^45,56–59^.

**Figure 5:**
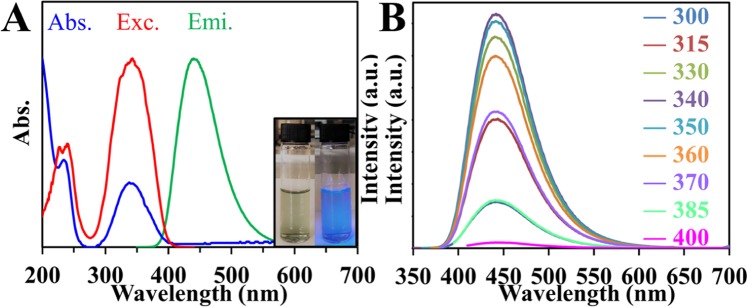
(**A**) UV-Vis Absorption (blue), Excitation (red) and Fluorescence emission (green) spectra of CDs. [Inset: Photographs of CDs in visible-light (left) and under UV-light (right)]. (**B**) Emission spectra of CDs when excited at different wavelengths. Excitation wavelengths are shown.

The optical properties of CDs were investigated using UV-Vis absorption and photoluminescence spectra of the prepared CDs (Fig. 5A). The UV−V is spectrum of the CDs in water displays an absorption peak peaked at 245 nm and a distinct band at 340 nm, which assign to π → π* transition of C=C and n → π* transition of C=O, correspondingly^20^. Consequently, excitation at 340 nm shows an emission spectrum at 450 nm consistent with blue PL. Shown as the inset is the light greenish CDs aqueous solution in daylight and the strong blue fluorescence emission when exposed by a 365 nm UV-light.

The optical properties were further investigated and the excitation-independent behavior was considered and labeled in (Fig. 5B). When the excitation wavelength is changed from 300 nm to 400 nm, all the emission is found at 450 nm. The emission is thought to occur only through the radiative transition of sp^2^ carbon, which will result in excitation independence due to the π → π* transition of the graphitic structure of the carbon cores. Excitation dependent emission is considered to be a common property of CDs for multicolored applications, but complex emission spectra are problematic to distinguish from one another in practical applications. Thus, CDs with only one emission are still very much anticipated^60,61^. The PL intensity increases with changing the excitation wavelength from 300 nm to 340 nm then gradually decreases from 340 nm to 400 nm.

The effects of some particular conditions such as various pH, different NaCl concentration, prolonged exposure to UV light and varied temperature on the stability of CDs were inspected.

**Figure 6:**
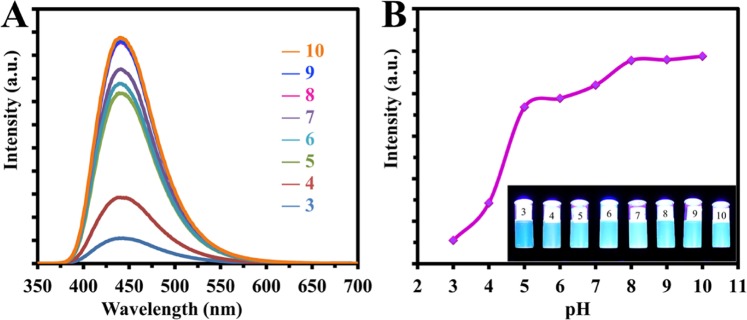
(**A**) Emission spectra of carbon dots in different pH (pH shown in the figure). (**B**) PL intensity of CD at different pH (inset is a photo of CD under UV-light in different pH).

As illustrated in Fig. 6A the influence of pH on the fluorescence intensity of the CDs was recorded. As shown in Fig. 6B, the fluorescence intensity increased as the pH values increased from 3 to 10. The pH values of these solutions were adjusted using dilute solutions of HCl or NaOH, then the fluorescence spectra of these solutions were recorded.

**Figure 7:**
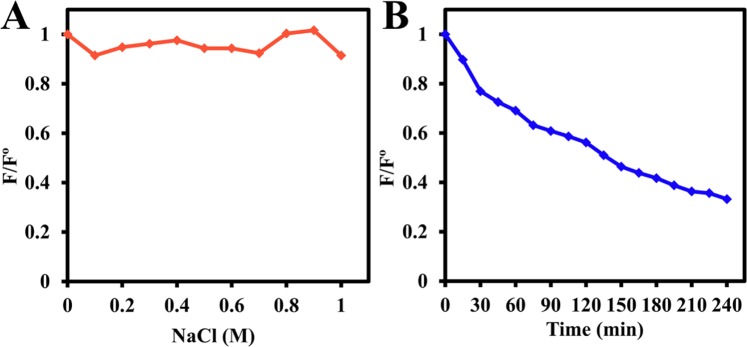
Effects of (**A**) Ionic strength, (**B**) Continuous irradiation by 6 watt UV lamp with a wavelength of 365 nm on CDs.

The fluorescence intensity of CDs was almost unchanged in different NaCl concentrations up to 1 M NaCl assigning for the stability of the CDs’ in the extreme ionic strength solution. However, the emission intensity decreased under continuous radiation for 4 hours, as in Fig. 7. A and B, respectively.

### CDs as a Nanothermosensor

**Figure 8:**
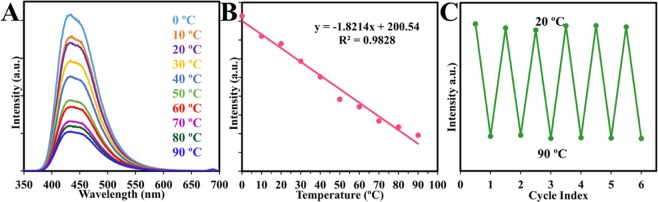
(**A**) Fluorescence spectra of CDs against different temperatures. (**B**) The linear relationship between fluorescence intensity and temperature. (**C**) Fluorescence intensity upon the cyclic switching of CDs under alternating conditions of 20 °C and 90 °C.

The fluorescence property of the CDs was investigated to study the influence of temperature. According to Fig. 8A, it was obvious that the fluorescence intensity decreased at higher temperatures and shows a well temperature-sensitive feature. The fluorescence intensity against temperature in the range (0–90 °C) showed a good linear relationship with a correlation coefficient (R^2^) of 0.9828 as shown in Fig. 8B which is a wider range than previous nanothermometers reported^7,14,36^. Interestingly, as shown in Fig. 8C the prepared fluorescent CDs exhibited outstanding reversibility and restorability of the fluorescence intensity when the temperature increased and decreased alternatively between 20 °C to 90 °C. In other words, the temperature does not destroy the surface fluorescent structure of the as-prepared CDs permanently. The thermal sensitivity calculated based on Fig. 8B was 1.8% °C^−1^, which is very good as compared with those reported in the literature^12,62^. Monitoring temperature in nano and micro spaces is very important for a deep understanding of the reaction mechanism and dynamics in nanosystems, as well as the behavior of a single living cell. Thus, modification of carbon nanodots will open a wide door to obtain a typical nanothermometer for various nano-applications.

The mechanism of the temperature-dependent property of carbon dots still is not well-established. However, Yang *et al*.^55^ attribute the thermal linear fluorescence quenching to the synergistic effects of abundant oxygen-containing functional groups and hydrogen bonds. The same experiments were conducted as Yang *et al*.^55^ performed. The first trial was the reduction of CDs using 0.1 M NaBH_4_ to obtain reduced CDs (r-CDs) then monitoring the thermal property of the CDs. On the second trial, dissolving the CDs in the ethanol (e-CDs) then investigating the thermosensing behavior. In both cases, the results of r-CDs and e-CDs were similar to the original results of CDs (Fig. S1, Supporting Information). Thus, our experiments rule out the synergistic effects of abundant oxygen-containing functional groups and hydrogen bonds. From here, the fluorescence decay with temperature can be attributed to the temperature-induced “energy traps” on the CDs surface, feasibly provoking energy transmission and quenching^3^.

### CDs as a sensor for Fe^2+^/Fe^3+^

The CDs were selectively quenched after the addition of ferrous and/or ferric ions, while other common metal ions and anions did not show any effect or their effect was negligible.

### Selectivity of CDs

**Figure 9:**
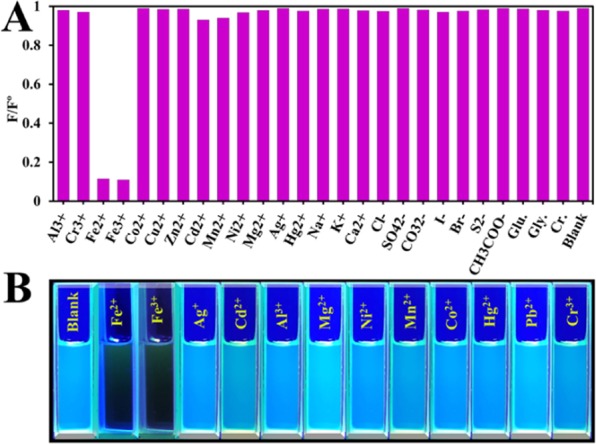
Fluorescence intensity of the CDs in the presence and absence of interferences. (**A**) Histogram showing the effect of cations and anions on the fluorescence intensity. (**B**) Digital photographs of the effect of cations and anions on the fluorescence intensity.

To verify that the CDs have selective response towards an analyte, we tested them against several ions as shown in (Fig. 9). Metal ions such as Na^+^, K^+^, Ca^2+^, Al^3+^, Cr^3+^, Fe^2+^, Fe^3+^, Co^2+^, Cu^2+^, Zn^2+^, Cd^2+^, Mn^2+^, Ni^2+^, Mg^2+^, Ag^+^, and Hg^2+^ were tested and other ions such as Cl^−^, SO_4_^2−^, CO_3_^2−^, I^−^, Br^−^, S^2−^, CH_3_COO^−^, glucose, Glycine and Creatinine were also tested only iron (Fe^2+^ and Fe^3+^) could quench the fluorescence intensity.

The interaction of the CDs and Fe^2+^ ions is pH-dependent. As the Fe^2+^ quenches the emission in pH values lower than 5, while above pH 5 precipitation occurs and that is assigned to the formation of iron hydroxides. (ksp of Fe(OH)_2_ = 8 × 10^−16^).

pH 5 is chosen throughout this work, as it is better than pH 4 or 3 because the CD has better intensity at higher pH values. Also for the study and analysis of Fe^3+^ ions same conditions (like pH) used as for Fe^2+^ ions because Fe^3+^ ions could quench the emissions from the CDs at almost all pH conditions without precipitate formation.

**Figure 10:**
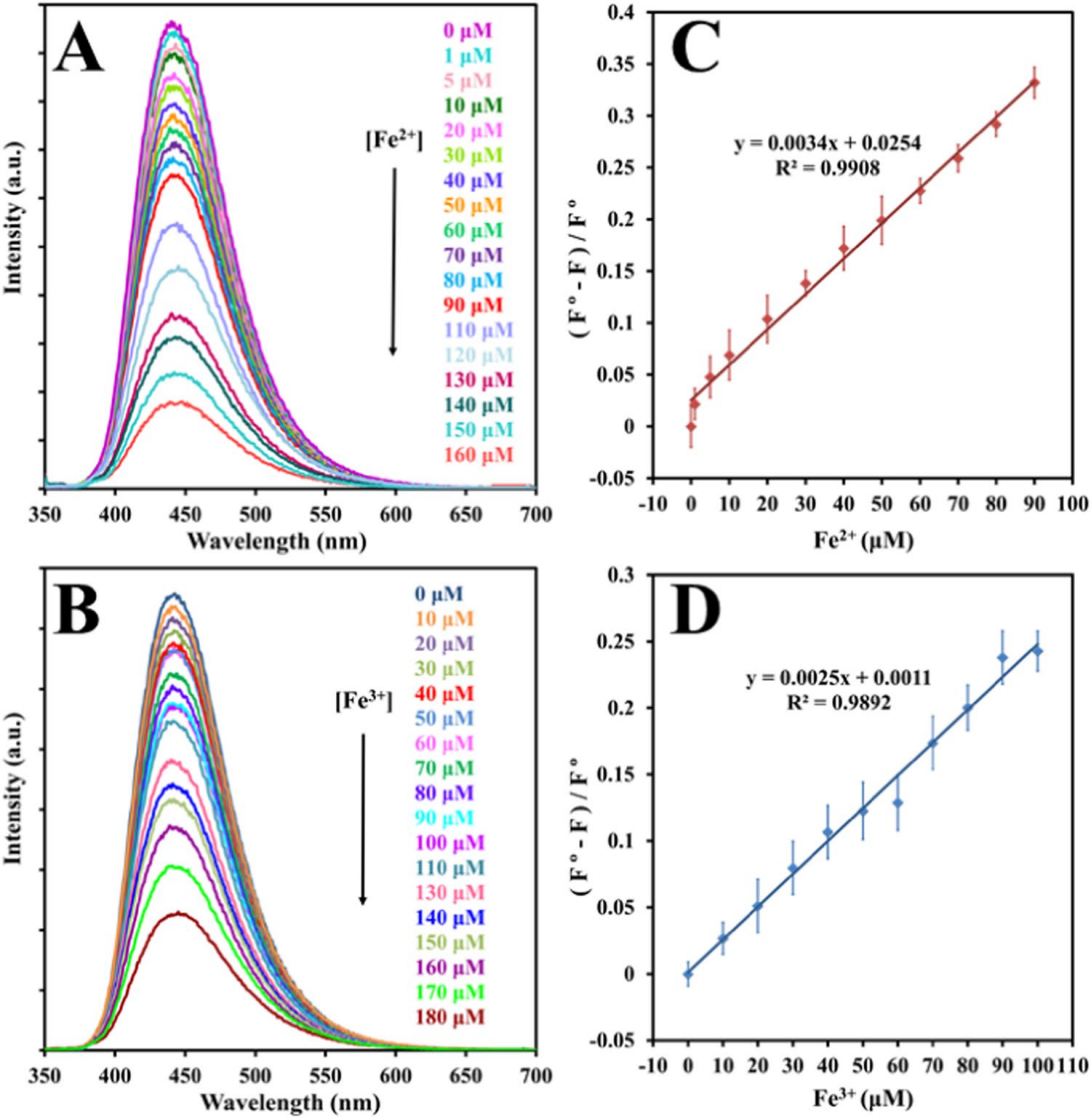
Fluorescence emission spectra of the CDs upon the addition of various concentrations of (**A**) Fe^2+^. (**B**) Fe^3+^. The relationship between the fluorescence quenching efficiencies of CDs and different concentrations of (**C**) Fe^2+^ ions (0, 1, 5, 10, 20, 30, 40, 50, 60, 70, 80, 90 μM), and (**D**) Fe^3+^ ions (0, 10, 20, 30, 40, 50, 60, 70, 80, 90, 100 μM).

To explore the quenching of CDs emission by each iron ions Fe^2+^and Fe^3+^ in aqueous solution, we mixed different concentrations of Fe^2+^ and Fe^3+^ with CDs separately. Figure 10A,B exposed the conversion of fluorescence intensity of the as-synthesized carbon dots in the presence of a varying concentration of Fe^2+^ and Fe^3+^ individually. It is confirmed plainly that the fluorescence could be quenched regularly with the increase of each ion. Accordingly shown in Fig. 10C,D, the quenching efficacy ((F°-F)/F°) exhibited a worthy linear correlation (R^2^ = 0.9908 for Fe^2+^ and R^2^ = 0.9892 for Fe^3+^) as opposed to concentration (in the range 0 to 100 μM), where F° and F are the fluorescence intensity at 340 nm in the absence and presence of iron ions, respectively. The limit of detection (LOD) was assessed to be 79 nM for Fe^2+^ and 107 nM for Fe^3+^, which was calculated according to a signal-to-noise ratio of S/N = 3. It is worth mentioning that only few articles have reported CDs that can detect both iron ions simultaneously, which makes this work one amongst only few that can detect Fe^2+^ ion as well as Fe^3+^ ion simultaneously. Compared with the other fluorescent CDs in literature shown in Table 1, the proposed B, N-co-doped-CDs showed comparable and/or better efficiency for the detection of Fe^3+^ and Fe^2+^ ions in terms of linear range and low LODs, indicating that it is a promising probe for sensing of iron ions.

**Table 1:** Comparison of proposed B,N-co-doped-CDs with other fluorescent CDs for Fe^3+^ and Fe^2+^ detection.

Iron ions	LOD	Linear Range	References
Fe^2+^	7.5 nM	0.020–10 µM	^17^
Fe^3+^	5.0 nM	0.010–10 µM	
Fe^2+^	20 nM	0–32 µM	^26^
Fe^3+^	35 nM	0–50 µM	
Fe^2+^	50 nM	0–200 μM	^65^
Fe^3+^	50 nM	0–200 μM	
Fe^2+^	100 nM	1–150 µM	^66^
Fe^3+^	100 nM	1–150 µM	
Fe^2+^	79 nM	0–90 μM	This work
Fe^3+^	107 nM	0–100 μM	

### Quenching mechanism by iron ions

In general, quenching of fluorescence refers to the interaction between a fluorophore and a quencher. Static quenching can occur as a result of the formation of a non-fluorescent ground state complex between the fluorophore and quencher. When this complex absorbs light it immediately returns to the ground state without emission of a photon^63^.

**Figure 11:**
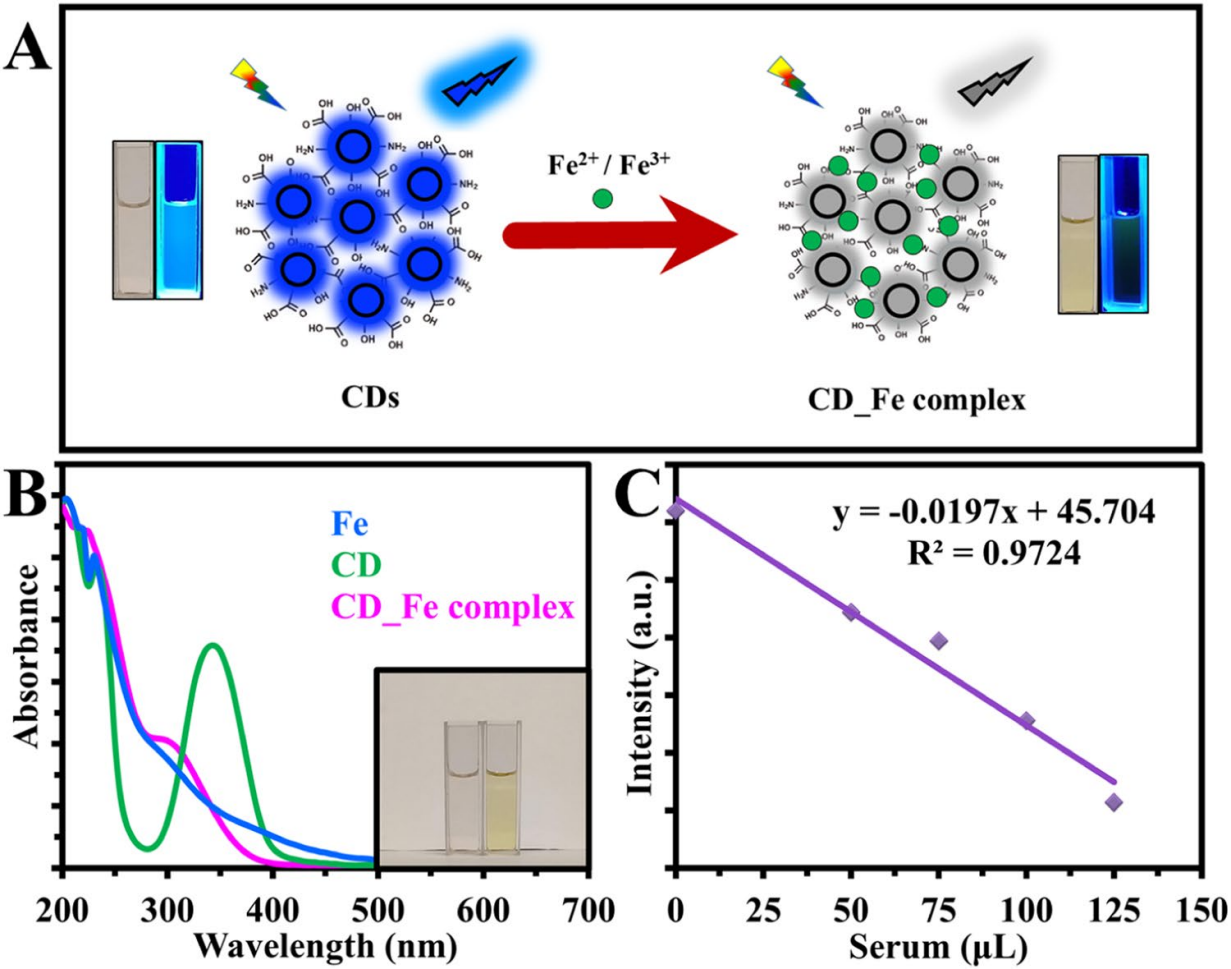
(**A**) Scheme showing the formation of CD_Fe complex and as a result quenching the emission by CDs. (**B**) UV-Vis absorption spectra of CDs, Fe^2+^, and CDs-Fe [inset is Photograph of CDs (left) and CDs_Fe (right)], (**D**) The linear calibration plot for Fe^3+^ detection in human blood serum.

It was confirmed that the fluorescence intensity of the carbon dots could be quenched significantly by Fe^2+^ and Fe^3+^. The fluorescence quenching caused by these ions was most likely due to static quenching rising from the formation of a stable non-fluorescence complex between surface functional groups of CDs and Iron ions. The color change of the solution was also an indication for the complex interaction between CDs and each Iron ions as is clarified in (Fig. 11A). To prove this plausible explanation, the UV-Vis absorption spectra of CDs, Fe^3+^, and CDs-Fe system were performed (Fig. 11B). The formation of the ground-state complex results in the change of the absorption spectrum of the CDs^63,64^.

### Application to real samples

Considering these necessary properties of as-prepared carbon dots, they were exploited as a particularly selective and sensitive fluorescent detector for Iron. The practical evaluation of this anticipated nanoprobe was established more by detecting Fe^2+^ in iron capsules, and Fe^3+^ in human blood serum with a standard addition technique.

The fluorimetric investigation with CDs was initially employed for Fe^2+^ ions in iron capsule solutions spiked with different concentrations of standard Fe^2+^ ions. The detection efficiencies of Fe^2+^ in spiked capsule samples were 102%, enlightening a dependable and practical method for Fe^2+^ detection.

Later on, to ensure the feasibility of CDs for diagnosing Fe in the clinic, we attempted to detect Fe^3+^ in serum samples. As shown in (Fig. 11C), the fluorescence intensity PL decreased with increasing the volume of deproteinized serum solution and there is a direct correlation between them. This confirmed that the suggested method was capable of monitoring Fe^3+^ in biological samples due to the notable selective property and admirable fluorescence of the CDs.

To determine the amount of Fe^3+^ in serum samples, a standard addition method using Fe(NO_3_)_3_ as the standard was carried out for two sera and the recoveries were 98% and 95% as compared to the results from clinical fully automatic biochemical analyzer (Cobas) (Table 2).

**Table 2:** Analysis of clinical samples (n = 5).

Sample	Cobas (μM)	CDs (μM)	Recovery%
Serum 1	17.3	17	98
Serum 2	12.5	11.9	95

## Conclusion

In summary, dual-functional luminescent B, N co-doped carbon nanodots were prepared and used for chemical and thermal detections. The thermo-sensor showed excellent recovery, good linearity, wide-range temperature sensing and reliable thermo-sensitivity at 1.8% °C^−1^. The fluorescence emission of CDs was selectively quenched by both ferric and ferrous ions. Fluorimetric analysis for each iron ions were exploited simultaneously. The fluorimetric investigation with CDs employed for Fe^2+^ ions in iron capsule solutions and Fe^3+^ in deproteinized serum samples. This confirmed that the suggested method was capable of detecting Fe^2+^ and Fe^3+^ in aqueous samples due to the notable selective property and high fluorescence of the CDs. The thermo-sensitivity and low LODs for Fe^3+^/Fe^2+^ ions suggest that the dual functional CDs is a novel fluorescent probe for thermo-chemical application. Our finding will open a door to tailor a variety of carbon nanodots to obtain reliable, sensitive, and low-cost nanothermometer.

## Supplementary information

Supplementary Information.
