# Supplementary material for: Dual functional highly luminescence B, N Co-doped carbon nanodots as nanothermometer and Fe3+/Fe2+ sensor
Source: Sci Rep. 2020 Feb 20;10:3028. doi: 10.1038/s41598-020-59958-5 (PMC7033239; doi:10.1038/s41598-020-59958-5)
Supplement: Supplementary file 1 — Supplementary Information. [file 41598_2020_59958_MOESM1_ESM.docx]

**Dual functional highly luminescence B, N Co-doped carbon nanodots as nanothermometer and Fe^3+^/Fe^2+^ sensor**

**Lazo Jaza Mohammad^1^ and Khalid M. Omer^1, 2^***

Department of Chemistry, College of Science, University of Sulaimani, Qliasan St, Sulaimani City, Kurdistan region, Iraq

*** Corresponding address E-mail:** [**khalid.omer@univsul.edu.iq**](mailto:khalid.omer@univsul.edu.iq)


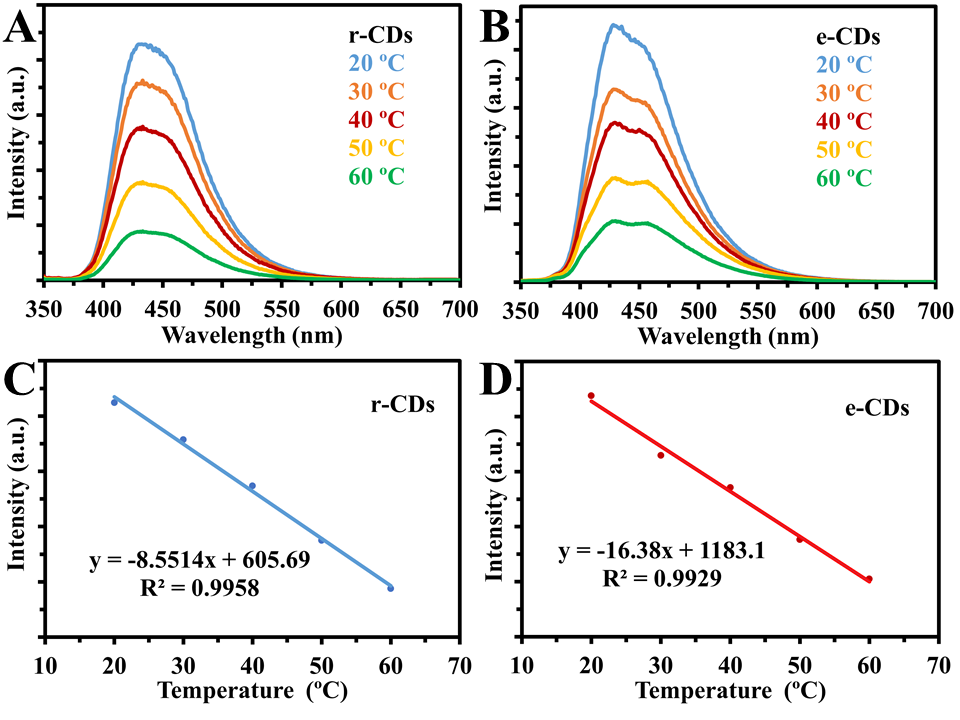


**Figure S1**. A. and B. Fluorescence spectra of reduced CDs (r-CDs) and CDs in ethanol (e-CDs) at Temperatures (20 to 60 ºC). C. and D. Linear correlation between fluorescence intensity and temperature (ºC) for r-CDs and e-CDs respectively.
